# Dr. John B. Lundy

**Published:** 1903-10

**Authors:** 


Dr. John B. Lundy, of Preston, Ont., a graduate of the University of Buffalo, 1851, died of paralysis, at his home, August 20, 1903, aged 77 years.

